# Preparation of Biocompatible Carboxymethyl Chitosan Nanoparticles for Delivery of Antibiotic Drug

**DOI:** 10.1155/2013/236469

**Published:** 2013-03-18

**Authors:** Liang Zhao, Bingya Zhu, Yunhong Jia, Wenjiu Hou, Chang Su

**Affiliations:** ^1^College of Pharmacy, Liaoning Medical University, Jinzhou 121000, China; ^2^College of Veterinary Medicine, Liaoning Medical University, Jinzhou 121000, China

## Abstract

*Objective*. To prepare biocompatible ciprofloxacin-loaded carboxymethyl chitosan nanoparticles (CCC NPs) and evaluate their cell specificity as well as antibacterial activity against *Escherichia coli* in vitro. *Methods*. CCC NPs were prepared by ionic cross-linking method and optimized by using Box-Behnken response surface method (BBRSM). Zeta potential, drug encapsulation, and release of the obtained nanoparticles in vitro were thoroughly investigated. Minimum inhibitory concentration (MIC) and killing profiles of free or ciprofloxacin-loaded nanoparticles against *Escherichia coli* were documented. The cytotoicity of blank nanoparticles and cellular uptake of CCC NPs were also investigated. *Results*. The obtained particles were monodisperse nanospheres with an average hydrated diameter of 151 ± 5.67 nm and surface of charge −22.9 ± 2.21 mV. The MICs of free ciprofloxacin and CCC NPs were 0.16 and 0.08 *μ*g/mL, respectively. Blank nanoparticles showed no obvious cell inhibition within 24 h, and noticeable phagocytosis effect was observed in the presence of CCC NPs. *Conclusion*. This study shows that CCC NPs have stronger antibacterial activity against *Escherichia coli* than the free ciprofloxacin because they can easily be uptaken by cells. The obtained CCC NPs have promising prospect in drug delivery field.

## 1. Introduction

Ciprofloxacin (CPFX), as one outstanding representative of the third generation of fluoroquinolone antimicrobials with broad antibacterial spectrum, strong antibacterial activity, and low effective inhibition concentration, has achieved numerous satisfactory results in clinical applications [1, 2]. The formulations of CPFX such as capsule, tablet, and injection have been widely used [3, 4]. However, these ordinary administration methods show that the highest bioavailability of the drug is approximately 52%. In addition, half-life of the useable CPFX is too short to realize prolonged drug release. Although many pathogens can be directly internalized into cells, most of drugs will be degraded by lysosome before killing bacteria. This phenomenon usually leads to the low intracellular drug concentration. The cells infected provide a huge storage in which pathogenic microbes and bacteria are propagated and amplified. Release of pathogenic bacteria often leads to recurrence of controlled infecting symptoms [5, 6]. Chitosan, as a good biodegradable, natural originated material, has been widely used as the sustained drug-release carrier in pharmaceutical field [7–9]. Carboxymethyl chitosan (CMC) is obtained by carboxylation reaction of amine group substitution by small alkyl groups. CMC is biocompatible and easy to degradation. Compared with chitosan, CMC has good solubility in water without aid of acid substance, thus avoiding the damage in pH sensitive drug antibacterial activity [10, 11]. Herein, in the present work, carboxymethyl chitosan nanoparticles were prepared by ionic cross-linking method to improve the cell uptake of drug efficacy and to prolong the acting time of the drug.

## 2. Experimental

### 2.1. Materials

Carboxymethyl chitosan (CMC) was obtained from Haixin Biological Product Co., Ltd (China), ciprofloxacin (98.7%) Zhejiang Jingxin Pharmaceutical Co., Ltd (China), and *Escherichia coli* ATCC-25922 was purchased from Nanjing Bianzhen Biotechnology Co., Ltd (China). Fluorescein isothiocyanate (FITC) and aluminum chloride were obtained from Sigma Chemicals (St Louis, USA). All the other used chemicals were of analytical grade.

### 2.2. Preparation of CCC NPs

0.05 g of carboxymethyl chitosan was dissolved in 50 mL deionized water, stirring and swelling overnight. Deionized water solution containing ciprofloxacin was dripped into the carboxymethyl chitosan solution and continued stirring for 2 h. Aluminum chloride reserve liquid (0.1 mg/mL) was prepared and filtrated through 0.45 *μ*m filter. CCC NPs were prepared by dropping AlCl_3_ reserve liquid quickly into the system at certain temperature and continuously stirred for 1 h until milk light appeared. CCC NPs collected were washed 3-4 times with deionized water and centrifuged at 16000 rpm for 20 min, freeze-drying to obtain powders.

### 2.3. Experiment Design and Data Processing

Box-Behnken design as a type of response surface method has been applied to pharmaceutical systems and particulate carriers. It provides detailed information to better understand potential interaction between various factors employed in pharmaceutical preparations for yielding the optimal formulation [12, 13]. Experiments were designed according to Box-Behnken response surface method and shown in Table 1. The selected factors were as follows: drug/carboxymethyl chitosan (*X*_1_), reacting temperature (*X*_2_), and the amount of AlCl_3_ (*X*_3_). CCC NPs were prepared, and their loading and encapsulation efficiency as response values were determined. Each response value was standardized between 0~1 as the normalized value by using Hassan method, and all normalized values were used to calculate the geometric mean and get general normalized value (OD) [14]. The formula was as follows:

(1) $$\begin{array}{c} {\text{OD}}_{i}={\left(d_{i}f_{i}\right)}^{1/2}\\ d_{i}=\frac{\left(Y_{i}-Y_{\min}\right)}{\left(Y_{\max}-Y_{\min}\right)}\\ f_{i}=\frac{\left(Z_{i}-Z_{\min}\right)}{\left(Z_{\max}-Z_{\min}\right)}, \end{array}$$

where *i*  represents the total test points from 1 to 17.

### 2.4. Characterization of CCC NPs

1~2 drops of CCC NPs solution were placed in a supporting copper film and stained using phosphotungstic acid. After drying, morphology of the nanoparticles was observed by transmission electron microscope (JEM-1200EX, Tokyo, Japan). CCC NPs solution was diluted with deionized water to the appropriate concentration and measured by Zetasizer (Nano ZS90, Malvern, UK) for determining hydrated swelling size distribution and surface charge.

### 2.5. Determination of Drug Loading and Encapsulation Efficiency

The total mass amount of CCC NPs was accurately weighed, and 2 mg sample of CCC NPs was dispersed in 10 mL 0.01 mol/L HCL under ultrasonic extraction for 0.5 h. The obtained mixture was filtered through a 0.45 *μ*m millipore filter for determining absorbance of the filtrate at 277 nm using a UV/Vis spectrophotometer (model 1601, Shimadzu, Japan). Loading efficiency (LE, %) and encapsulation efficiency (EE, %) were calculated using (2):

(2) $$\begin{array}{c} \text{LE}\left(\%\right)=\frac{\text{amount}\;\;\text{of}\;\;\text{CPFX}\;\;\text{in}\;\;\text{NPs}}{\text{weight}\;\;\text{of}\;\;\text{NPs}}\times 100\\ \text{EE}\left(\%\right)=\frac{\text{amount}\;\;\text{of}\;\;\text{CPFX}\;\;\text{in}\;\;\text{NPs}}{\text{Initially}\;\;\text{added}\;\;\text{CPFX}}\times 100. \end{array}$$

### 2.6. Investigation of Drug Release Behavior

Accurate weighed 10 mg CCC NPs wrapped in a dialysis bag (spectrum, USA) were placed in 30 mL phosphate buffer solution (pH = 7.4). The temperature was maintained at 37.0 ± 0.5°C and the stirring speed at 100 rpm. 3 mL of buffer solution was taken out at 0.5, 1, 2, 3, 4, 6, 8, 10, 12, and 24 h, and 3 mL fresh phosphate buffer solution (pH = 7.4) was added to maintain the release medium volume at the same time. Samples were filtered through 0.45 *μ*m filter and analyzed spectrophotometrically at 277 nm.

### 2.7. Antibacterial Determination of CPFX and CCC NPs against *Escherichia coli* ATCC-25922

CPFX and CCC NPs were diluted with LB culture medium to 10.24, 5.12, 2.56, 1.28, 0.64, 0.32, 0.16, and 0.08 *μ*g/mL (8 levels) calculated by the concentration of CPFX and 100 *μ*L from each level was added into 96-well plate. 100 *μ*L of bacteria liquid was added into wells containing different concentration of CPFX, and the number of *Escherichia coli* ATCC-25922 in well was 1 × 10^6^ CFU/mL. LB culture medium without CPFX was also used to inoculate bacteria under the same method as blank control. The sample was incubated at 37°C and removed after 20 h, then determining the lowest concentrations of no bacterial growth as minimum inhibitory concentration (MIC).

Each 1.5 mL solution of free CPFX and CCC NPs diluted with LB culture medium to 0.16 *μ*g/mL of CPFX was added to test tubes followed by the addition of 1.5 mL bacteria liquid in which concentration of bacterial colony was 2 × 10^6^ CFU/mL. Samples were cultured in the incubator and drug-free tube was set (liquid 1.5 mL + medium 1.5 mL) as blank control. 10 *μ*L of samples was removed at 1, 2, 4, 6, and 8 h to count colony. Curve was made from logarithm of colony number against different culturing time.

### 2.8. Cell Viability Assays

SMMC-7221 liver carcinoma cells were seeded into each well of a 96-well cell culture plate. After culturing for 24 h, nanoparticles without CPFX were added into the exposed cells, and concentrations calculated by CMC were 0.1, 0.2, 0.4, 0.8, 1.2, 1.8, and 2.4 mg/mL. After culturing for 24 h, 96-well cell culture plate was taken out. 10 *μ*L four methyl thiazolyl tetrazolium salt (MTT) was added to 10 *μ*L culture medium from each well and continued culturing. Cultural supernatant was discarded and 100 *μ*L (sodium dodecyl sulfate) SDS-HCl was placed in each well overnight. ELISA meter (model 2550, Biorad, USA) was applied and the absorbance value was measured at 570 nm.

### 2.9. In Vitro Cellular Uptake of CCC NPs

SMMC-7221 liver carcinoma cells (2 mL, 5 × 10^5^/mL) were seeded to 6-well plates and were cultured overnight. CCC NPs encapsulating FITC were added into the medium and incubated for 8 h. After centrifugation the supernatant was discarded and cells in well were washed with ice-cold PBS for 3 times. Finally cells were observed by fluorescence microscope.

## 3. Results

### 3.1. The Result of RSM

The result in regression variance analysis and significance test in the model is shown in Table 2. Lack of fit in the model was 298.89, *P* < 0.0001, and showed extremely significant difference among models. Model determination coefficient *R*_adj_^2^ value was 0.9176 and showed that there was linear relationship between the dependent variables and all independent variables. The variance analysis of regression model also displayed that *X*_1_, *X*_2_ were significant, and the interaction term *X*_1_ *X*_3_, a square term *X*_1_^2^, demonstrated high significance. *F* value of the model is 20.79 and the probability of *P* < 0.0005, which indicated that the model had a high fitting degree. A multiple linear regression equation on the relationship between selected factors and general normalized value OD is established as

(3) OD=+0.48+0.077X1−0.055X2  −7.500E−003X3−0.040X1X2  −0.26X1X3+0.075X2X3  −0.28X12+0.085X22+0.060X32.

### 3.2. Response Surface Analysis

**Figure 1:**
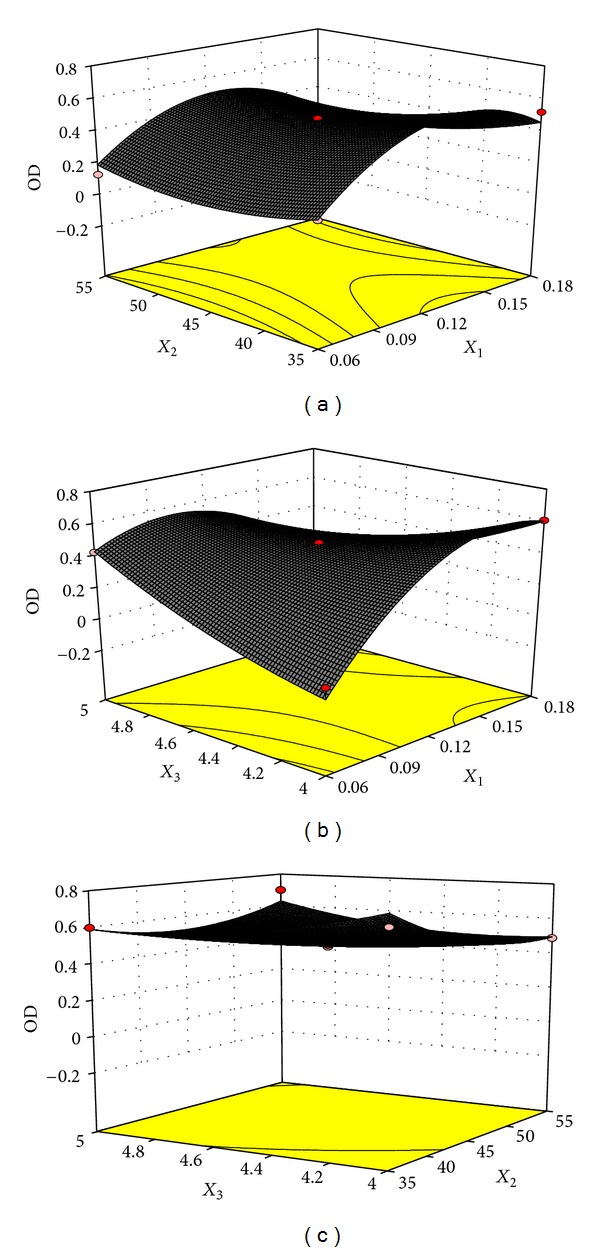
Effects of *X*_1_-*X*_2_ (I), *X*_1_-*X*_3_ (II), and *X*_2_-*X*_3_ (III) on OD (*X*_1_: mass ratio between drug and carboxymethyl chitosan, *X*_2_: reacting temperature, *X*_3_: amount of AlCl_3_).

3D figures of response surface designed by using OD as the dependent variable and two actors as variables are shown in Figure 1. OD value was increased with the increase of the ratio of drug/carboxymethyl chitosan. When the ratio of drug/carboxymethyl chitosan was 0.16, the OD value was the highest. After that, OD value was reduced with the increase of the ratio of drug/carboxymethyl chitosan. In terms of reacting temperature, OD value showed first decrease and then an upward trend in the range of 35–55°C. As the effect of amount of AlCl_3_ on OD value was not significant, any amount should be applied between 4.0 and 5.0 mg. The optimal formulation of CCC NPs by using RSM was concluded as follows: when the ratio of drug/carboxymethyl chitosan was 0.16, reacting temperature was 35°C, and the amount of AlCl_3_ was 4.0 mg, the predicted OD was 88.7%. Drug loading efficiency was (76.0 ± 4.31)%, drug encapsulation efficiency was (92.3 ± 7.21)%, and OD was (83.8 ± 5.11)%. Model predicting results were close to actual data, demonstrating that established model was reliable.

### 3.3. Appearance of CCC NPs

**Figure 2:**
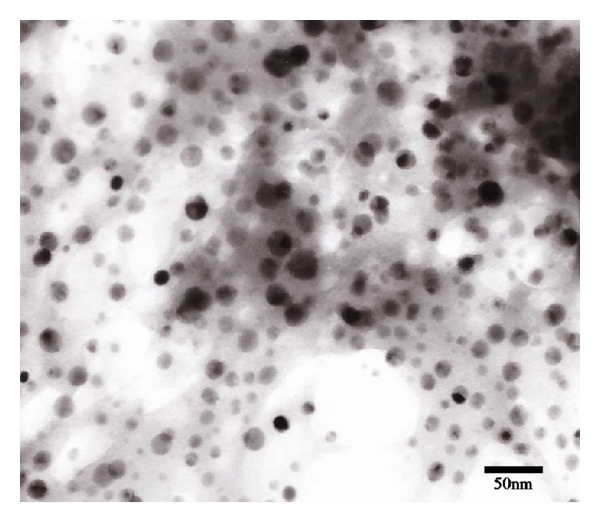
Transmission electron microscopy image of CCC NPs.

Figure 2 shows that under the optimized condition, the obtained CCC NPs are spherical pellets with good monodispersity and homogenous particle size at average hydrated diameter of 151 ± 5.67 nm and surface charge of −22.9 ± 2.21 mV measured by Zetasizer.

### 3.4. In Vitro Drug Release

**Figure 3:**
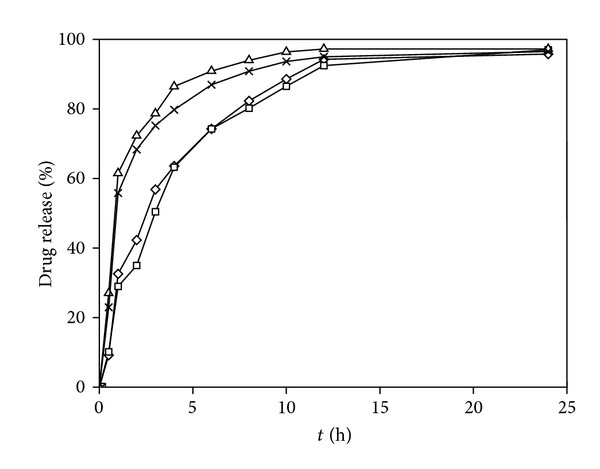
Release profiles of CPFX from nanoparticles (F10 = Δ, F12 = ×, F9 = ⋄, F7 = □) (*n* = 3).

Figure 3 shows that CCC NPs have obvious sustained drug release behavior. About 9%–27% of total drug was released rapidly at first 0.5 h and more than 95% released in 24 h. Results of release in vitro by using release kinetics models were fitted, trying to find the best drug release model.

The correlation coefficient (*R* ^2^) was closer to 1, the fitting effect was better. As shown in Table 3, release rate and time had good relationship fitted by using equations among which double exponential equation having the highest value of *R* ^2^ was the best to describe the drug release behavior. Drug was released rapidly in the initial stage and restricted into the medium at significantly slower drug release rate in the next period. 

### 3.5. Antibacterial Activity against *Escherichia coli* ATCC-25922 In Vitro

**Figure 4:**
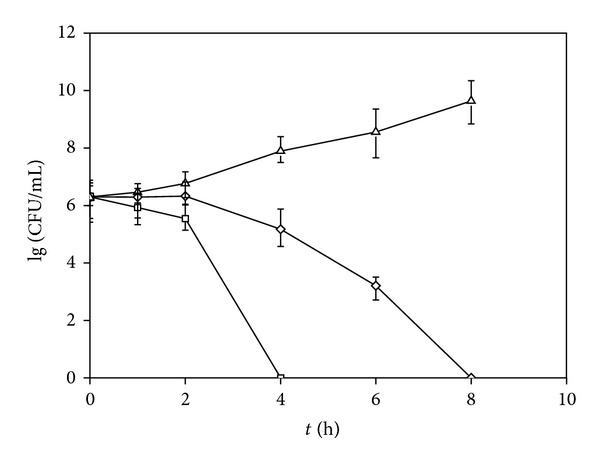
Killing profiles of free CPFX or CCC NPs against *Escherichia coli* ATCC-25922 (control = Δ, free CPFX = ⋄, CCC NPs = □) (*n* = 3).

The MIC of CPFX and CCC NPs was 0.16 and 0.08 *μ*g/mL. Compared with free ciprofloxacin, antibacterial activity of ciprofloxacin loaded in nanoparticles was increased by 2 times. Killing curve of free or ciprofloxacin in nanoparticles against *Escherichia coli* ATCC-25922 is shown in Figure 4. Sterilization time of free CPFX and CCC NPs at the same concentration was 8 h and 4 h. It demonstrated that compared with free CPFX, CCC NPs tended to kill more bacteria and shortened the time of sterilization.

### 3.6. Cell Viability Assays of Drug-Free Nanoparticles

**Figure 5:**
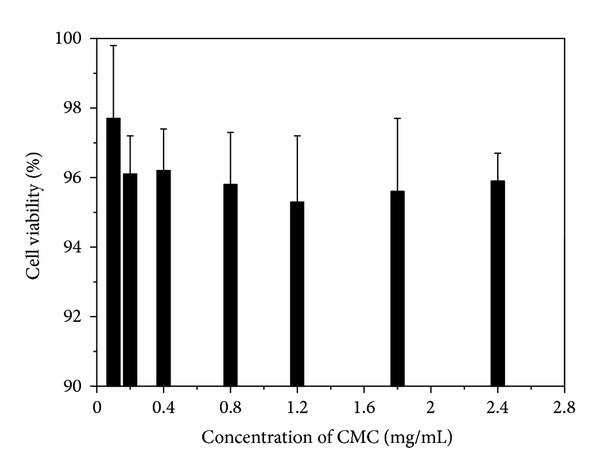
In vitro viability of SMMC-7221 cells cultured with different concentrations of CMC for 24 h (*n* = 3).

Drug-free nanoparticles were incubated with cells for 24 h, and MTT assay was used to evaluate the cell viability at different concentrations of CMC. It can be seen from Figure 5 that drug-free nanoparticles with different concentrations of CMC showed no obvious cell inhibition within 24 h, and the cell survival rate in all samples was above 95%. This demonstrates that CMC is biocompatible at the dosage of 0.1–2.4 mg/mL.

### 3.7. In Vitro Cellular Uptake of CCC NPs

**Figure 6:**
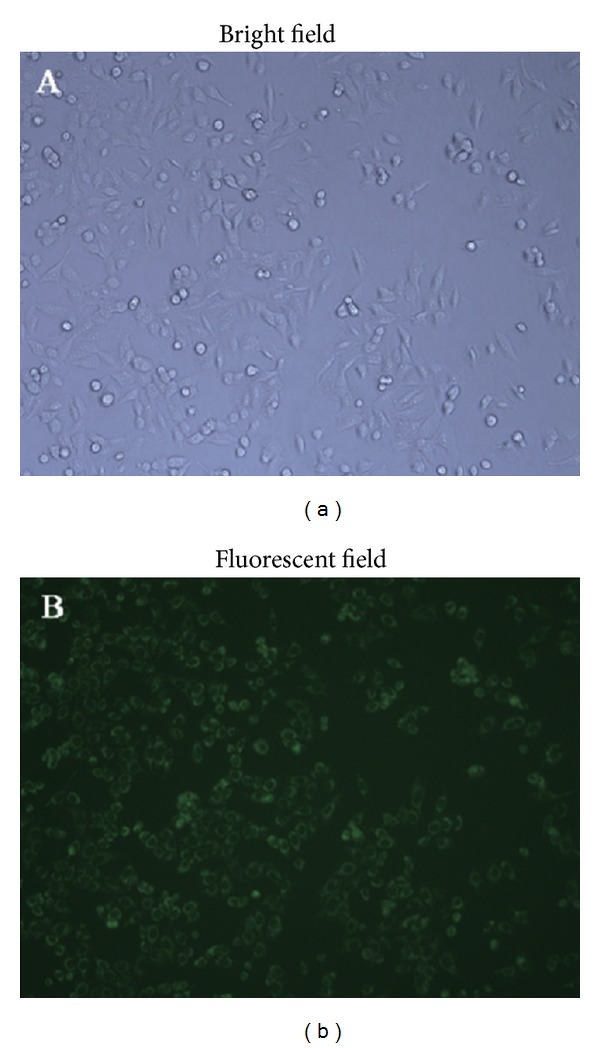
Evaluation of cellular uptake (internalization). FITC-labeled CCC NPs were incubated with SMMC-7221 liver carcinoma cells for 8 h at 37°C.

Uptake of CCC NPs on cells is shown in Figure 6. After cells and FITC-labeled CCC NPs were incubated for 8 h, FITC showed high fluorescence intensity in cells, which demonstrated that FITC-labeled CCC NPs were internalized into the cells. As CCC NPs show higher uptake into cells, they may be good carriers for delivery of antibiotic drug.

## 4. Discussion

Compared with formulation optimization by using uniform design method which aims at investigating effects of single factor on the preparation of nanoparticles, Box-Behnken response surface method not only ensures measurement accuracy, but also studies interacting effects among different actors [15]. The results show that the predicted OD value of the optimizing formulation is similar with actual result, implying that the established mathematical model by using Box-Behnken response surface method has a good predictability [16, 17].

CCC NPs showed different releasing pattern in the different stage. It implied that drugs absorbing on the surface of nanoparticles detached into the releasing medium rapidly, resulting in the burst release of drug in the initial stage. As more holes were formed by further degradation and dissolution of CMC into the medium, drug moved through inside pores in nanoparticles slowly and fell into the medium by diffusion.

Endocytosis is considered to be the main mechanism of cellular uptake of CCC NPs. Nanoparticles were recognized with the mediation of the opsonin and boud with cell plasma membrane. Part of the plasma membrane invaginated to form vesicles in which nanoparticles were wrapped and were separated from plasma membrane into the interior of the cell. When vesicles further contacted with lysosome, drug in vesicles was released into cytoplasm by enzymatic hydrolysis.

## 5. Conclusion

Formulation for ciprofloxacin-loaded carboxymethyl chitosan nanoparticles was optimized by using Box-Behnken response surface method. Under the optimized condition, the average value of hydrated diameter was (151 ± 5.67) nm, zeta potential was (−22.9 ± 2.21) mV, drug loading efficiency was (76.0 ± 4.31)%, drug encapsulation efficiency was (92.3 ± 7.21)%, and OD was (83.8 ± 5.11)%. Antibacterial activity of ciprofloxacin loaded in nanoparticles was increased by 2 times. The carrier material had good biocompatibility, and no significant cytotoxicity was observed after incubating ciprofloxacin-free nanoparticles with SMMC-7221 liver carcinoma cells. Furthermore, CCC NPs showed obvious cellular uptake, thereby improving the antimicrobial drug permeability.

## Figures and Tables

**Table 1:** Design scheme and response values.

Formulation code	*X*_1_^a^	*X*_2_^b^	*X*_3_^c^	LE% (*Y*)^d^	EE% (*Z*)^e^	OD^f^
1	0.18	45.00	4.00	5.76	58.27	0.61
2	0.12	55.00	5.00	3.71	85.52	0.70
3	0.06	45.00	5.00	5.89	48.29	0.43
4	0.18	55.00	4.50	5.33	43.48	0.27
5	0.18	35.00	4.50	6.24	51.68	0.52
6	0.12	35.00	4.00	5.08	68.29	0.69
7	0.18	45.00	5.00	3.36	39.25	0
8	0.12	45.00	4.50	4.85	53.42	0.47
9	0.06	55.00	4.50	1.53	58.38	0.13
10	0.12	45.00	4.50	4.88	53.42	0.47
11	0.06	45.00	4.00	1.31	49.30	0
12	0.12	45.00	4.50	4.76	54.65	0.48
13	0.12	55.00	4.00	4.19	58.44	0.49
14	0.12	45.00	4.50	4.55	55.21	0.48
15	0.06	35.00	4.50	2.01	55.08	0.22
16	0.12	35.00	5.00	4.00	70.23	0.60
17	0.12	45.00	4.50	4.66	55.04	0.48

^a^Drug/carboxymethyl chitosan, weight/weight; ^b^reacting temperature, °C; ^c^added amount of AlCl_3_; ^d^loading efficiency; ^e^encapsulation efficiency; ^f^general normalized value.

**Table 2:** Analysis of variance in RSM.

Source	Sum of squares	df	Mean square	*F* value	*P* value probe > *F *
Model	0.72	9	0.080	20.79	0.0003
*X*_1_	0.048	1	0.048	12.45	0.0096
*X*_2_	0.024	1	0.024	6.27	0.0408
*X*_3_	4.500*E* − 004	1	4.500*E* − 004	0.12	0.7428
*X*_1_ *X*_2_	6.400*E* − 003	1	6.400*E* − 003	1.66	0.2388
*X*_1_ *X*_3_	0.27	1	0.27	70.05	<0.0001
*X*_2_ *X*_3_	0.022	1	0.022	5.83	0.0465
*X*_1_^2^	0.32	1	0.32	82.79	<0.0001
*X*_2_^2^	0.030	1	0.030	7.79	0.0269
*X*_3_^2^	0.015	1	0.015	3.86	0.0901
Residual	0.027	7	3.860*E* − 003		
Lack of fit	0.027	3	8.967*E* − 003	298.89	<0.0001
Pure error	1.200*E* − 004	4	3.000*E* − 005		

Cor total	0.75	16			

*P* < 0.001 is highly significant; *P* < 0.05 is significant.

**Table 3:** Regression results of equations in vitro release from CCC NPs.

	Zero-order		First-order		Higuchi		Ritger-Peppas		Double exponential
Code	*Q*_*t*_ = *Kt*		ln(1 − *Q*_*t*_) = *Kt*		*Q*_*t*_ = *Kt* ^1/2^		*Q*_*t*_/*Q*_*∞*_ = *Kt* ^*n*^		1 − *Q*_*t*_ = *Ae* ^*αt*^ + *Be* ^*βt*^
	*R* ^2^	*K*	*R* ^2^	*K*	*R* ^2^	*K*	*R* ^2^	*n*	*R* ^2^
F7	0.850	0.072	0.990	0.221	0.971	0.288	0.888	0.638	0.995
F9	0.873	0.072	0.992	0.204	0.977	0.287	0.934	0.639	0.988
F10	0.598	0.060	0.945	0.283	0.831	0.266	0.801	0.337	0.998
F12	0.637	0.061	0.937	0.234	0.858	0.265	0.811	0.374	0.999

Noted “*Q*_*t*_” and “*Q*_*∞*_” are accumulative release rates of CPFX in CCC NPs at time “*t*” and “*∞*”.
